# Live imaging and quantitative analysis of *Aspergillus fumigatus* growth and morphology during inter-microbial interaction with *Pseudomonas aeruginosa*

**DOI:** 10.1080/21505594.2020.1827885

**Published:** 2020-10-05

**Authors:** Sebastian Wurster, Gabriele Sass, Nathaniel D. Albert, Hasan Nazik, Eric Déziel, David A. Stevens, Dimitrios P. Kontoyiannis

**Affiliations:** a Department of Infectious Diseases, Infection Control and Employee Health, The University of Texas M.D. Anderson Cancer Center, Houston, TX, USA; b California Institute for Medical Research, San Jose, CA, USA; c INRS-Centre Armand-Frappier Santé Biotechnologie, Laval, Quebec, Canada; d Division of Infectious Diseases and Geographic Medicine, Department of Medicine, Stanford University School of Medicine, Stanford, CA, USA

**Keywords:** Mixed infection, intermicrobial interaction, live imaging, morphogenesis, iron metabolism, *Pseudomonas*, *Aspergillus*

## Abstract

*Pseudomonas aeruginosa* (PA) and *Aspergillus fumigatus* (AF) chronically colonize the airways of patients with cystic fibrosis or chronic immunosuppression and mutually affect each other’s pathogenesis. Here, we evaluated IncuCyte time-lapse imaging and NeuroTrack^TM^ (NT) analysis (Wurster et al., 2019, mBio) as a toolbox to study mycelial expansion and morphogenesis of AF during interaction with PA. Co-incubation of AF with supernatant filtrates of wild-type (WT) PA strains strongly inhibited hyphal growth and branching. Consonant with prior metabolic studies, pyoverdine-deficient PA mutants had significantly attenuated inhibitory capacity. Accordingly, purified PA products pyoverdine and pyocyanin suppressed mycelial expansion of AF in a concentration-dependent way. Using fluorescence-guided tracking of GFP-AF293 mycelia during co-culture with live WT PA cells, we found significant inoculum-dependent mycelial growth inhibition and robust precision of the NT algorithm. Collectively, our experiments position IncuCyte NT as an efficient platform for longitudinal analysis of fungal growth and morphogenesis during bacterial co-infection.

## Introduction

Polymicrobial infections have increasingly become a focus of interest in infectious diseases research as inter-kingdom interplay of pathogens can mutually affect their virulence, susceptibility to antimicrobial therapy, and interactions with host immune surveillance [1]. In particular, *Aspergillus fumigatus* (AF) and *Pseudomonas aeruginosa* (PA) frequently co-colonize the airways of patients with cystic fibrosis, chronic bronchiectasis, or chronically immunocompromised patients and compete for nutrients in their ecological niche [2]. The mutually antagonistic relationship of AF and PA is, to a large extent, mediated by extracellular PA products, including toxins and siderophores [2]. Specifically, PA metabolites with iron-binding capacity, such as pyoverdine or pyocyanin, inhibit growth, biofilm formation, and metabolism of AF [2–4].

Whereas most of these studies used conventional measurements such as growth inhibition or changes in metabolism [2], there is a need for robust and efficient experimental systems that specifically assess morphogenesis, a key feature of fungal virulence [5], in the context of bacterial interactions. Previous work revealed that dirhamnolipids released by PA induce ultrastructural modifications of AF hyphae, resulting in altered mycelial morphology and reduced growth of AF [6]. However, currently available modalities to study mycelial expansion, hyphal morphology, and biofilm formation, such as fluorescence or electron microscopy [6–8] and spectroscopy techniques [9], have low throughput and are time-intensive. In addition, most currently employed assays for inter-kingdom interaction studies [2–4,6–9] present endpoint analyses that are not suitable for efficient longitudinal monitoring.

Inspired by morphological and functional similarities of neuronal networks and fungal mycelia [10], we have recently proposed the IncuCyte time-lapse microscopy platform and its NeuroTrack^TM^ (NT) image processing module as an efficient and reliable tool to study the expansion kinetics and viability of mycelial trees [11]. In this study, we hypothesized that NT analysis could present an appealing platform to quantitatively analyze mycelial morphology and expansion in the context of intermicrobial interaction. To that end, we validated the NT technology for the assessment of PA and AF interplay and implemented fluorescence-guided real-time tracking of AF mycelia in the presence of live PA cells.

## Materials & methods

### Fungal strains and culture

Conidia of *A. fumigatus* AF10 and a GFP-expressing AF293 strain were collected from mature colonies grown on yeast extract agar for 48–72 h at 37 °C. Conidia were passed through a 40 µm cell strainer, washed twice in sterile saline, and counted with a hemocytometer.

### Bacterial culture and generation of filtrates

Planktonic culture filtrates of wild-type (WT) PA strains and PA14 mutants (Table S1) were prepared as detailed previously [12]. Briefly, PA was quantitated, suspended in RPMI 1640 medium (Sigma-Aldrich) either supplemented or not with 25 µM FeCl_3_, and grown for 24 h at 37°C. Bacterial cultures were filtered (0.22 μm) after the growth period to remove bacterial cells and debris.

### Co-culture set-up

Aliquots of conidial suspensions (200 conidia in 100 µl RPMI medium) were dispensed per well of a 96-well flat-bottom plate. RPMI medium (100 µl, control), purified PA metabolites pyoverdine (1–10 µM) or pyocyanin (50–500 µM) (both: Sigma-Aldrich), filtrates of WT PA strains and siderophore loss-of-function mutants, or viable PA cells were added. For live cell co-culture, PA/AF ratios of 0.01 to 100 were tested in 10-fold serial dilutions.

### IncuCyte imaging

Well plates were incubated and imaged for 18 h at 37 °C in the IncuCyte ZOOM HD/2CLR time lapse microscopy system (Sartorius) equipped with an IncuCyte ZOOM 10 x PLAN FLUOR objective (Sartorius) as previously described [11]. Phase images were acquired hourly for all experiments. For live cell co-culture using AF293-GFP, green fluorescence images were obtained hourly with an acquisition time of 400 milliseconds. NT image processing algorithms were applied using our published parameters for *A. fumigatus* and AF293-GFP [11].

### Data analysis

Raw data for NT endpoints “neurite (hyphal) length” and “branch points” were exported to Microsoft Excel and further processed using GraphPad Prism v8. For each well and NT endpoint, the area-under-the-curve (AUC) was determined, considering the first 18 h of culture. AUCs were normalized to a medium or “AF only” control (= 1.0). Unless indicated otherwise, 3 or 4 replicates with independently prepared bacterial and fungal inocula and/or filtrates were assessed. Significance tests and levels are specified in the figure legends. Coefficients of variation (CVs) for relative AUCs values were calculated by dividing standard deviations by arithmetic means.

## Results

**Figure 1.**
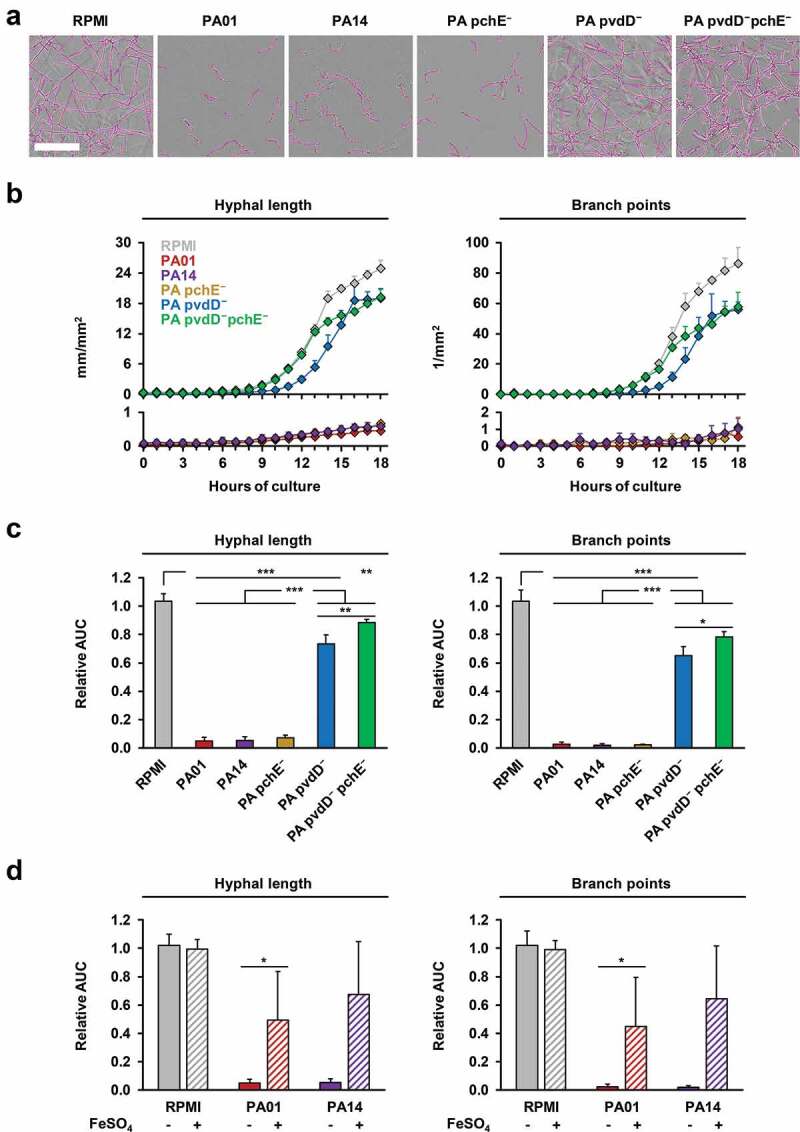
Comparative inhibitory activity of culture filtrates of WT and siderophore-deficient *P. aeruginosa* strains on mycelial expansion of *A. fumigatus.*

To validate the IncuCyte NT technology for studies of AF-PA interplay, we tested the differential inhibitory impact of well-characterized WT PA strains and PA mutants [2] on the expansion and morphogenesis of AF mycelia. Compared to the RPMI control, co-incubation of AF10 with culture filtrates of WT PA strains PA01 and PA14 strongly inhibited hyphal growth and branching (Figure 1(a,b)). Mean relative AUC values for NT endpoints in cultures of AF conidia exposed to filtrates of either WT PA strain were 0.05 for hyphal length and 0.02 for branch points (Figure 1(c), p < 0.001). In line with prior metabolic studies [2], the inhibitory effect was largely attenuated when using supernatants of a pyoverdine-deficient PA mutant (PA14 *pvdD^−^*) or a pyoverdine-pyochelin double PA mutant (PA14 *pvdD^−^pchE^−^*) (p < 0.001), whereas pyochelin deficiency alone (PA14 *pchE^−^*) had no impact on the inhibitory capacity of PA (Figure 1(a–c)).

AF10 conidia were co-cultured with filtrates of two WT PA strains (PA01 and PA14), three siderophore loss-of-function mutants (PA14 *pvdD^−^, pchE^−^*, and *pvdD^−^pchE^−^*), or RPMI medium (control) in the IncuCyte ZOOM time lapse microscopy system. Four independent experiments with technical triplicates in each run were performed. (a) Representative images of AF mycelia after 16 h of co-culture with RPMI or the studied PA filtrates. Pink overlays indicate mycelial structures recognized by the NT algorithm. Scale: 250 µm. (b) Development of hyphal length and branch points was tracked for a period of 18 hours. Mean results from one representative experiment and intra-assay standard deviations are shown. (c) AUC values (0–18 h) of hyphal length and branch point numbers in wells exposed to filtrates or RPMI (blinded) were normalized to an internal, unblinded medium control. Mean relative AUCs and inter-replicate standard deviations are shown. One-way ANOVA with Tukey’s multiple comparison test was used for significance testing. (d) Relative AUC values (0–18 h) of hyphal length and branch point numbers of AF10 exposed to PA01 and PA14 culture filtrates generated using either regular (solid columns) or iron-supplemented RPMI (cross-hatched columns, 25 µM FeCl_3_). RPMI either supplemented or not with FeCl_3_ was used as the respective controls. The two-sided Student’s t-test was applied to each ± FeCl_3_ pair for significance testing. * p < 0.05, ** p < 0.01, *** p < 0.001.

Furthermore, we compared mycelial growth inhibition by WT PA filtrates depending on iron availability, as iron is a source of competition between AF and PA in their *in vivo* environment [2]. FeCl_3_ supplementation of the RPMI control did not affect the NT endpoints of AF (relative AUC, 0.99–1.02, Figure 1(d)), suggesting that baseline iron levels in our growth medium were not a limiting factor for mycelial expansion. In contrast, iron supplementation considerably attenuated the inhibitory effect of PA culture filtrates on AF growth and morphogenesis, as indicated by an increase in relative hyphal length and branch point AUC values from 0.05 to 0.49–0.68 (p = 0.03–0.07) and 0.02 to 0.45–0.64 (p = 0.03–0.08), respectively (Figure 1(d)).

**Figure 2.**
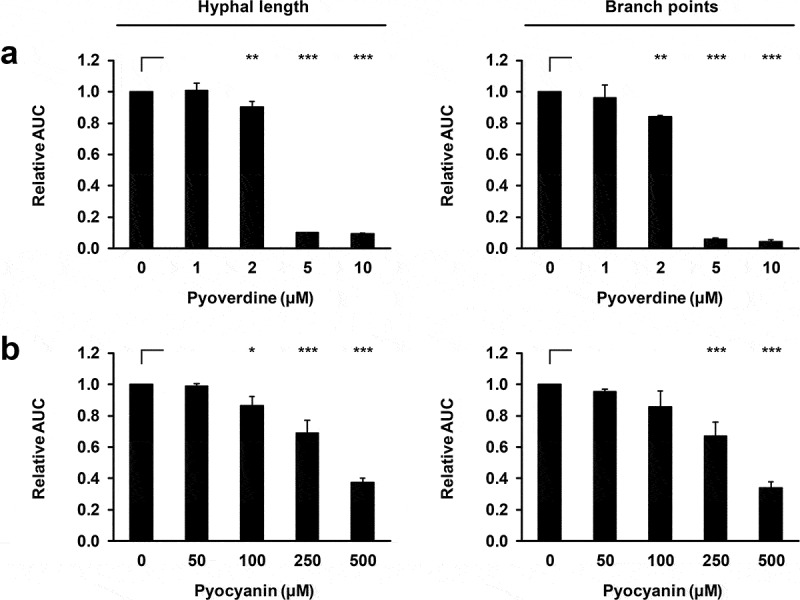
*P. aeruginosa* products pyoverdine and pyocyanin concentration-dependently inhibit mycelial expansion and branching of *A. fumigatus.*

To underscore the critical role of iron in our NT assay, we exposed AF to different concentrations of purified iron-sequestering PA products pyoverdine and pyocyanin that are known inhibitors of AF growth and metabolism [2–4,13]. Pyoverdine at concentrations between 2 and 5 µM resulted in a sharp inhibition of hyphal growth (p < 0.001, Figure 2(a)). Consonant with an earlier report [13], gradually increasing inhibitory activity of pyocyanin across a broader range of concentrations (50–500 µM) was seen (Figure 2(b)).

(a) AF10 conidia were incubated in RPMI supplemented or not with different concentrations of pyoverdine or pyocyanin and imaged in the IncuCyte ZOOM time-lapse microscopy platform. Mean relative AUC values and standard deviations (technical triplicates) of hyphal length and branch point numbers are shown. One-way ANOVA with Dunnett’s multiple comparison test (versus unsupplemented RPMl) was used for significance testing. * p < 0.05, ** p < 0.01, *** p < 0.001.

**Figure 3.**
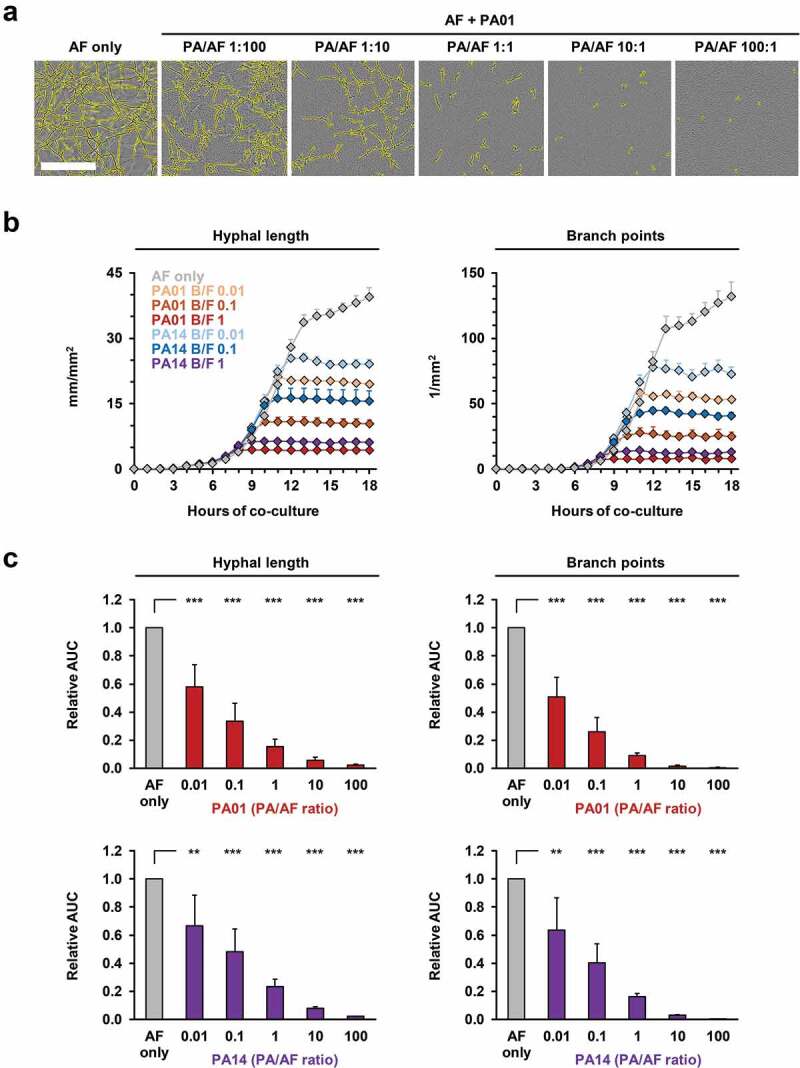
Fluorescence-based NT analysis facilitates efficient longitudinal tracking of inoculum-dependent inhibition of *A. fumigatus* growth and morphogenesis during co-culture with live *P. aeruginosa* cells.

To facilitate real-time image analysis of mycelial proliferation and morphogenesis during co-culture with different concentrations of viable PA cells, we used a GFP-expressing AF293 strain and a fluorescence-based NT algorithm. Cells of either PA WT strain suppressed both hyphal growth and branch point formation in an inoculum-dependent fashion (Figure 3(a), Figure S1A). Although initial inhibition of AF morphogenesis at low PA inoculums was rather moderate, a time-dependent increase in inhibition was seen, with a plateau reached after 8–10 h of co-culture (Figure 3(b), Figure S1B), whereas PA cells continued to proliferate (**Movie S1**). Even at PA/AF ratios as low as 0.01, a 33–49% reduction in mycelial elongation and hyphal branching was seen. Relative AUCs further dropped to 0.09–0.23 at a 1:1 ratio (p < 0.01) and a 100:1 PA/AF ratio completely abolished fungal germination (p < 0.001, Figure 3(c)). No interference of bacterial cells or auto-fluorescence with the image analysis algorithm and no deviations in autofocus accuracy were observed (**Movie S1**). Median intra-assay CVs for hyphal length and branching in bacterial co-culture were 7.8% and 11.8%, respectively, and inter-replicate CVs were even lower (Table S2).

Conidia of a GFP-expressing *A. fumigatus* AF293 strain (200 per well) were incubated in sterile RPMI (“AF only”) or co-cultured with WT PA01 and PA14 cells suspended in RPMI medium. Initial ratios of bacterial versus fungal cells (B/F ratios) ranged from 0.01 (2 PA cells per well) to 100 (2 x 10^4^ PA cells per well). (a) Representative images of AF mycelial morphology after 16 hours of co-culture with PA01 depending on the PA/AF ratio. Yellow overlays indicate mycelial structures recognized by GFP-based NT analysis. Scale: 250 µm. (b) Development of hyphal length and branch points depending on the PA strain and inoculum added. Mean results from one representative plate and intra-assay standard deviations based on technical triplicates are shown. (c) AUC values (0–18 h) of hyphal length and branch point numbers in co-culture wells were normalized to the AF only control. Mean relative AUCs based on 3 independent plates and inter-replicate standard deviations are shown. One-way ANOVA with Dunnett’s multiple comparison test (versus AF only) was used for significance testing. ** p < 0.01, *** p < 0.001.

## Discussion

Efficient techniques for longitudinal visualization and analysis of morphological endpoints could expand our understanding of the complex inter-kingdom relationships of fungal and bacterial pathogens competing for the same ecological niches. To that end, we applied the IncuCyte NT live imaging and analysis platform to AF co-cultures with purified PA products, filtrates of PA mutants, and viable PA cells. NT analysis has a number of crucial advantages compared with conventional endpoint assays of fungal proliferation, including an improved longitudinal resolution of phenotypic parameters, capacity to assess multiple well plates concurrently, and a high degree of automation based on pre-programmed routines, resulting in decreased hands-on time [11,14].

To evaluate the suitability of the platform for bacterial and fungal co-culture studies, we first verified the system’s capacity to recapitulate known inhibitory effects of siderophore-deficient PA mutants and purified iron-binding PA metabolites on AF mycelial formation. Iron is a central nutrient for AF [15], and the antifungal activity of PA has been tightly linked to the production of iron-sequestering metabolites, interfering with fungal iron availability [2–4]. The PA siderophore pyoverdine has been suggested as the key inhibitory molecule, especially in low iron environments [2,3], while more recent studies have stressed the dominance of pyocyanin as the main PA competitive molecule when iron is plentiful [16]. Our data suggest that NT analysis has excellent statistical power to detect the differential inhibitory activity of PA mutants and concentration-dependent effects of purified PA products. Therefore, the NT technology would be appealing as an efficient screening assay for bacterial metabolites or mutants interfering with fungal morphogenesis and expansion.

Despite the explosive growth of PA in RPMI medium resulting in considerable turbidity of the wells at high PA/AF ratios, no changes to the processing algorithms for fungal mono-culture or immune cell challenge [11] were needed to maintain high accuracy of NT analysis during co-culture with viable PA cells. Furthermore, no interference of bacterial auto-fluorescence with the image analysis algorithm was encountered. This observation is consonant with a previous report that excitation and emission spectrums of PA, but also other bacterial pathogens of potential interest for future studies (e.g. *Escherichia coli* or *Staphylococcus aureus*), do not overlap with the IncuCyte fluorescence channels [17]. Technical variation of NT endpoints in bacterial co-cultures slightly exceeded that in fungal monocultures, likely due to the additional variation introduced by the bacterial seeding step and potential asynchrony of inhibitory PA effects on AF mycelia. However, the reproducibility of NT endpoints was well within recommended ranges for microbiological cellular bioassays [18]. Importantly, reproducibility remained excellent even at very low PA inocula. Therefore, our titratable system can avoid or delay overgrowth of mycelia by PA cells during biofilm formation, a common issue of experimental *in vitro* systems for co-culture studies [19].

In contrast to NT analysis, most metabolic methods to track fungal proliferation are severely confounded by the presence of viable bacteria. For example, widely used colorimetric tetrazolium-based assays to quantify metabolically active fungal biomass require the use of bacterial filtrates [3] or extensive post-processing of mycelia after recovery from bacterial co-culture [20], as residuals of viable bacteria would metabolize tetrazolium [21] and cause false-positive results.

As with all assays, there are limitations of NT analysis to consider. Differential activity of PA mutants against preformed AF biofilms and planktonic cultures has been described [3,7]. While IncuCyte NT assessment is powerful to longitudinally study germination and early mycelium formation in planktonic cells, the method performs poorly in resolving morphological features of preformed biofilms as the autofocus-guided image acquisition procedure does not provide control of the focusing depth by the user [11]. Another limitation inherent to the GFP-based live cell co-culture approach is the need for sufficiently bright and stable fluorescent labeling approaches and/or availability of fluorescent fungal mutants, a circumstance that could complicate the assessment of clinical mold isolates. In addition, the effects described pertain directly to intermicrobial interactions in liquid media, mimicking, for example, what would occur in bronchial secretions. However, the dynamics and effects of mutations and microbial products might be different in studies of microbes on solid substrates, such as agar, mimicking epithelial or endothelial surfaces. As a specific limitation of the present study, the experiments were performed under normoxic conditions, whereas hypoxia is a common feature in a cystic fibrosis microenvironment, affecting growth characteristics and inter-microbial interactions of the studied pathogens [22]. Future studies with an IncuCyte system placed in oxygen-controlled environments could, therefore, present an exciting research direction.

In summary, the IncuCyte NT technology provides a new, powerful toolbox for intermicrobial interaction studies, facilitating real-time analysis of key morphological endpoints during live cell co-culture with unparalleled longitudinal resolution and robust technical precision. Furthermore, employing a commercially available platform and published processing algorithms [11], NT analysis could contribute to improved standardization of inter-microbial co-culture experiments across different laboratories. Paired with its capacity to accurately track antifungal drug effects in a spectrum of clinically relevant molds and yeasts [11], NT analysis could have important translational implications for studies of antifungal drug activity. Of note, Briard and colleagues showed that quorum-sensing controlled PA rhamnolipids have synergistic activity with azole antifungals against AF [6]. Providing a rapid and reliable platform to test these interdependencies during live cell co-culture, IncuCyte NT could refine drug screening studies in inter-kingdom infection settings such as mixed fungal and bacterial pneumonia in cancer patients or poly-microbial wound infections.

## Supplementary Material

Supplemental Material
