# Supplementary material for: Live imaging and quantitative analysis of Aspergillus fumigatus growth and morphology during inter-microbial interaction with Pseudomonas aeruginosa
Source: Virulence. 2020 Oct 5;11(1):1329–36. doi: 10.1080/21505594.2020.1827885 (PMC7549912; doi:10.1080/21505594.2020.1827885)
Supplement: Supplemental Material [file KVIR_A_1827885_SM7886.pdf]

Live imaging and quantitative analysis of *Aspergillus fumigatus* growth and morphology during inter-microbial interaction with *Pseudomonas aeruginosa*

Supplementary Materials

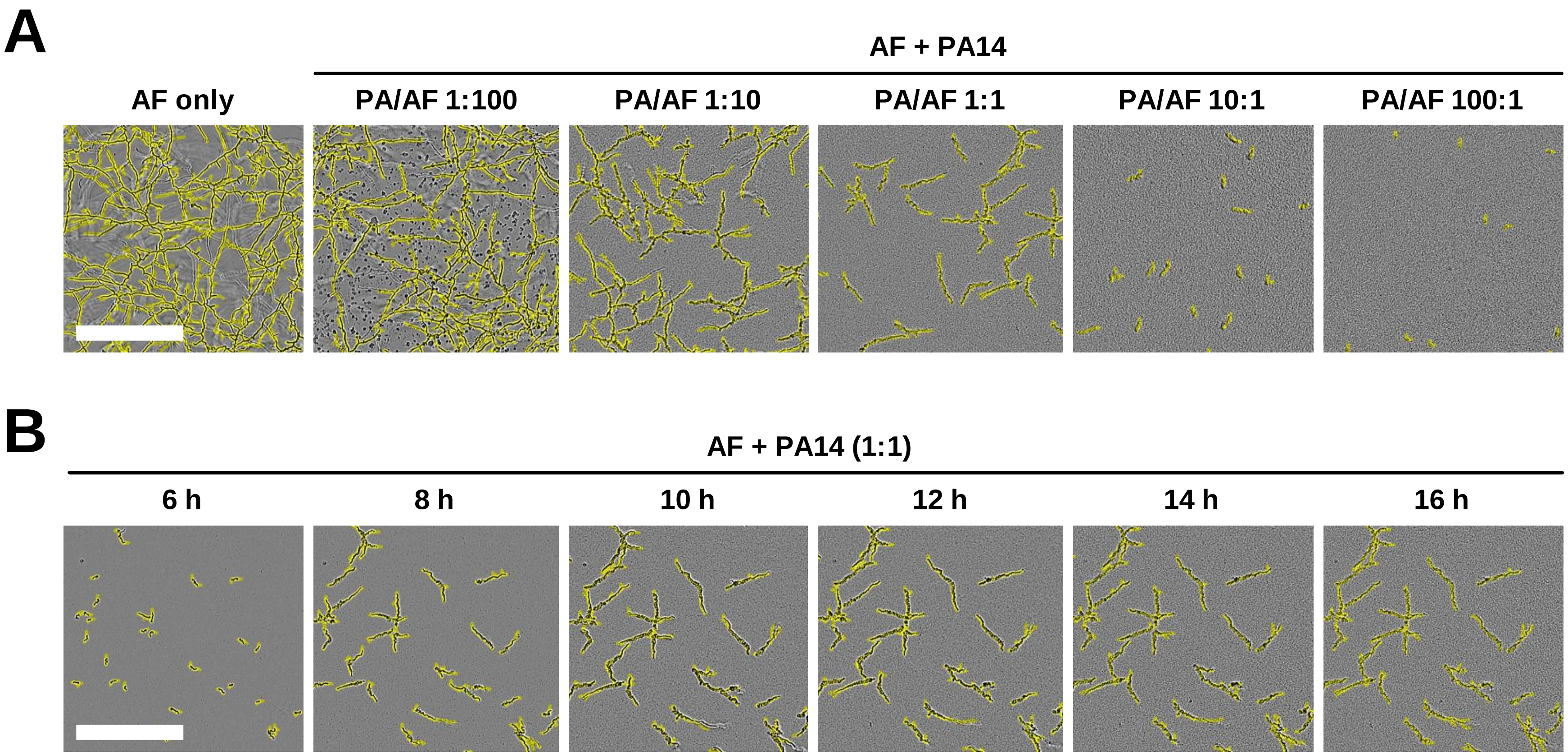

Figure S1. Representative images of time- and inoculum-dependent inhibition of *A. fumigatus* growth and morphogenesis by *P. aeruginosa* (PA14). Conidia of a GFP-expressing AF293 strain (200 per well) were incubated in sterile RPMI ("AF only") or co-cultured with wild-type PA14 cells suspended in RPMI medium. Initial ratios of bacterial versus fungal cells (B/F ratios) ranged from 0.01 (2 PA cells per well) to 100 (2 x 10^4 PA cells per well). (A) Representative images of mycelial morphology after 16 hours of co-culture depending on the PA/AF ratio. (B) Series of images from a 1:1 co-culture of GFP-AF293 and PA14 after different culture periods. Yellow overlays indicate mycelial structures recognized by GFP-based NT analysis. Scale: 250 µm.

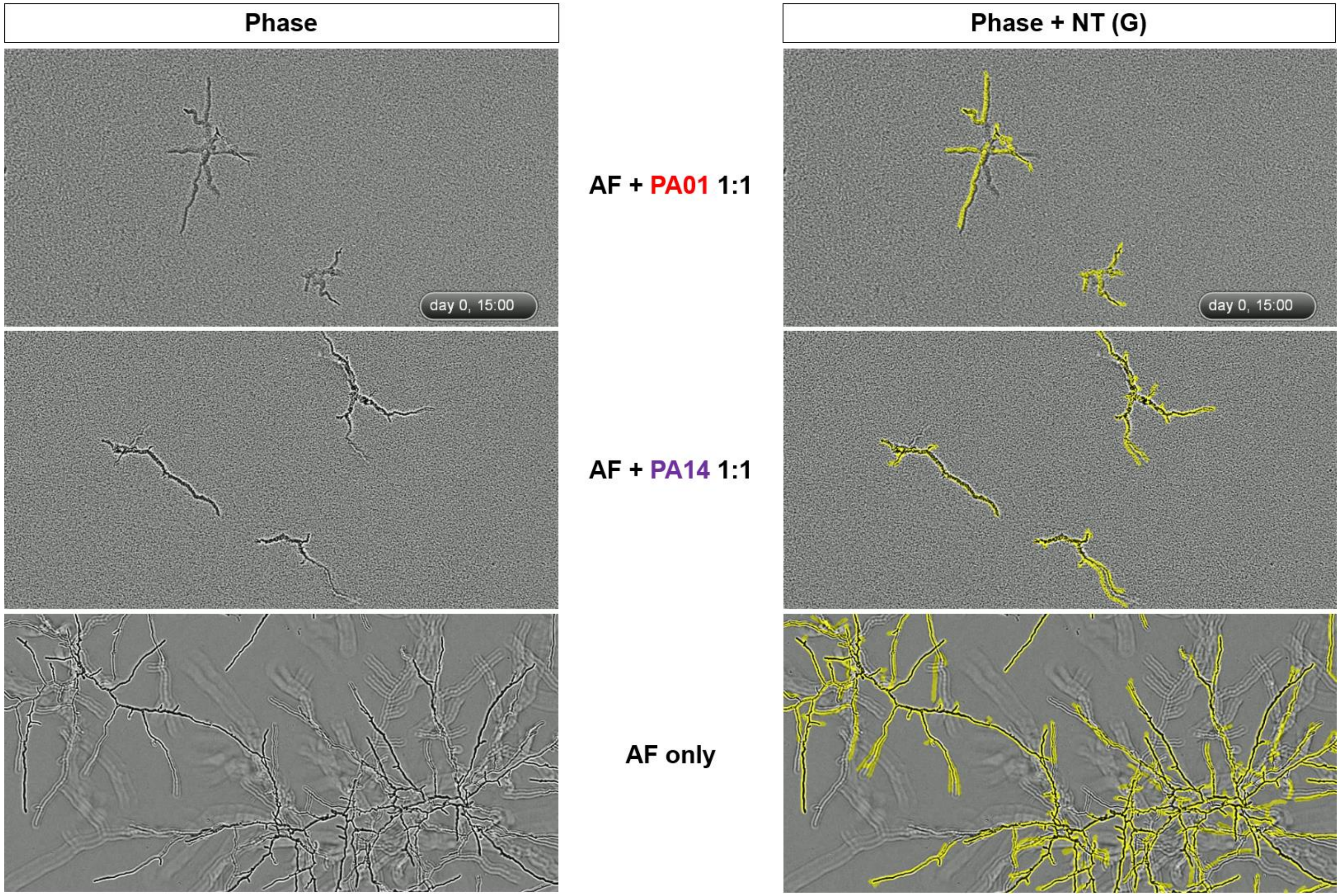

Movie S1. GFP-based tracking of GFP-AF293 mycelium during co-culture with PA01 and PA14.

Conidia of GFP-expressing *A. fumigatus* reference strain AF293 (200 conidia/well) were incubated in RPMI medium or co-cultured with 200 cells of PA01 or PA14. Image stacks were obtained by IncuCyte ZOOM time-lapse microscopy for 18 h at 37 ˚C. Yellow overlays indicate mycelial structures recognized by GFP-based NT analysis. [A representative still image from the movie is shown above]

Table S1. Source of *Pseudomonas aeruginosa* strains and mutants

Acronym	Characteristics	Source/reference
PA01	Wild-type isolate	ATCC 15692
PA14	Wild-type isolate, parental strain of the siderophore mutants used in this study	Rahme et al. 1995 Science, 268:1899-1902.
PA14 *pvdD*$^{-}$	Loss of pyoverdine (siderophore)	Liberati et al. 2006. Proc Natl Acad Sci U S A, 103:2833–2838.
PA14 *pchE*$^{-}$	Loss of pyochelin (siderophore)	Liberati et al. 2006. Proc Natl Acad Sci U S A, 103:2833–2838.
PA14 *pvdD*$^{-}$*pchE*$^{-}$	Pyoverdine-pyochelin double-siderophore mutant	Sass et al. 2018. J Bacteriol, 200(1): e00345-17.

Table S2. Reproducibility parameters for hyphal length and branch point AUCs in AF/PA live cell co-culture.

**Co-culture condition**	**Total hyphal length**			**Hyphal branch points**		
	Mean relative AUC	Median intra-assay CV[a]	Inter-replicate CV[b]	Mean relative AUC	Median intra-assay CV[a]	Inter-replicate CV[b]
**AF only**	**1.00**	**4.0 %**	n/a	**1.00**	**5.2 %**	n/a
+ PA01 B/F 0.01	0.58	7.9 %	0.1 %	0.51	6.9 %	2.3 %
+ PA01 B/F 0.1	0.34	8.0 %	4.5 %	0.26	12.7 %	7.4 %
+ PA01 B/F 1	0.16	4.3 %	0.4 %	0.09	9.4 %	2.8 %
+ PA01 B/F 10	0.06	4.3 %	3.4 %	0.02	15.6 %	8.6 %
+ PA01 B/F 100	0.02	12.9 %	2.5 %	< 0.01	18.7 %	10.9 %
+ PA14 B/F 0.01	0.67	7.7 %	2.5 %	0.64	10.5 %	3.8 %
+ PA14 B/F 0.1	0.48	11.7 %	4.5 %	0.40	11.8 %	5.2 %
+ PA14 B/F 1	0.23	6.6 %	3.4 %	0.16	11.7 %	5.8 %
+ PA14 B/F 10	0.08	6.3 %	9.7 %	0.03	9.8 %	8.4 %
+ PA14 B/F 100	0.02	14.9 %	1.3 %	< 0.01	29.8 %	19.9 %
**Median CV for AF/PA co-culture**		**7.8 %**	**3.0 %**		**11.8 %**	**6.6 %**

[a] Intra-assay CVs were determined based on technical triplicates on the same plate.

[b] Inter-replicate CVs were determined based on two plates prepared using the same fungal and bacterial stock solutions, with technical triplicates on each plate.

Abbreviations: AUC = area under the curve, CV = coefficient of variation, B/F = bacteria/fungus ratio, AF = *Aspergillus fumigatus*, PA = *Pseudomonas aeruginosa*, n/a = not applicable (no variation as relative AUC value of the AF only condition is set of 1.00 by default.
